# Evaluation of the Global Lung Function Initiative 2012 reference values for spirometry in an Iranian population

**DOI:** 10.1038/s41598-022-17306-9

**Published:** 2022-07-27

**Authors:** Leyla Sahebi, Besharat Rahimi, Mamak Shariat, Seyyed Hosein Mousavy

**Affiliations:** 1 Maternal, Fetal and Neonatal Research Center, Family Health Research Institute, Tehran University of Medical Sciences, Tehran, Iran; 2 Advanced Thoracic Research Center, Tehran University of Medical Sciences, Tehran, Iran; 3 Municipality of Tehran, Tehran, Iran

**Keywords:** Health care, Medical research

## Abstract

Spirometry is an important measurement in detecting and monitoring of chronic obstructive pulmonary disease. The validity of the multi-ethnic Global Lung Function Initiative 2012 (GLI-2012) spirometric norms have been debated in some countries. The aim of the present study was to evaluate the applicability of the GLI reference norms in the Iranian population. A cross-sectional study was performed on 622 healthy non-smoker population (204 males and 418 females, age range: 4 ± 82 years) between July 16 and August 27, 2019 in Iran. Z-scores for spirometric data [FEV_1_ (forced expiratory volume in 1 s), FVC (forced vital capacity) FEV_1_/FVC, and FEF_25–75%_ (forced expiratory flow averaged over the middle portion of FVC)] were calculated. According to the agreement approved, a mean Z-score outside the range of ± 0.5 was considered clinically significant. The mean (SD) Z-score values of FEV_1_, FVC, FEV_1_/FVC and FEF_25–75%_ were 0.44 (1.21), 0.49 (1.14), 0.11 (1.03), and − 1.13 (0.99) in males and 0.61 (1.14), 0.89 (1.26), 0.17 (0.88) and − 0.49 (0.96) in females, respectively. The Z-score of FEV_1_/FVC was below the lower limit of normal (LLN) in 3.43% of men and 2.01% of women (in ≥ 21 years), while these values were significantly higher in people under 21 years old (46.2% in boys and 40.0% in girls). The GLI reference values are not perfect for the Iranian population, especially in children below 10 years old. The use of the GLI reference values was appropriate in population above 21 years; however, they would overestimate the prevalence of airway obstruction in individuals below 21 years.

## Introduction

Spirometry is a pivotal screening test for the diagnosis of patients with obstructive lung disease. The main spirometry values include forced vital capacity (FVC), and forced expiratory volume (FEV1)^1,2^. These measurements are generally compared with the percentage predicted values. The predicted data are acquired from a healthy non-smoker standard population^3,4^. However, the predicted normal data change widely from different sources leading to biased results^5–7^. This bias can be avoided by using the sex, age, height, and ethnicity-specific Z-score^8^.

In 2012, the Global Lung Initiative (GLI-2012) released spirometric norms derived from data collected from 72031 healthy individuals aged 3–95 years^8^. The GLI-2012 equations provided sex, age, height, and ethnic-specific reference equations as well as the lower limit of normal (LLN) values for spirometry.

In pulmonary function testing, the fifth percentile of all normal values (a Z-score of − 1.64) is defined as the lower limit of normal (LLN). Spirometry indices at the LLN would be observed in only 1 in 20 (5%) normal populations^9^.

The fit of the GLI-2012 norms has been tested and some countries approved them for their use to interpret the spirometry results, for example, in the Australasian^10^, Norwegian^1^, German^11^, and French^12^ populations. On the other hand, the GLI-2012 norms seem unsuitable for clinical use in the Swedish^13^, Finnish^14^, Brazilian^15^, Malaysian^16^, and Chinese populations^17^. Some countries, including Iran, have not standardized the GLI-2012 equations^8^, although external validation of the GLI-2012 norms is recommended^7,8^. Moreover, the applicability of the norms should be evaluated in other parts of the world to verify their suitability in these regions. No study has evaluated the applicability of the GLI-2012 norms in the Iranian population.

The present study aimed to evaluate whether the GLI reference values apply to the Iranian population.

## Method

### Design

A cross-sectional study was performed in Tehran, the capital of Iran between July 16 and August 27, 2019. This article was the result of a research project approved by the National Institute for Medical Research Development (NIMAD) (code: 978931, 2019/05/28) and the Ethics Committee (code: IR.NIMAD.REC.1398.257). All methods were performed in accordance with the relevant guidelines and regulations. Written consent was obtained from all participants.

### Source population and sampling

The source population of this study was recruited from Tehran population presenting to health houses affiliated with Tehran Municipality. The sampling method was the randomized clustering method, and at least two health houses in each municipality district were selected (44 centers). The individuals who presented to health centers received an explanation about the study; then, they were asked to refer family members (3–95 years) if they were willing to join. In the second step, a short meeting was held with the household members or the household head in relation to the purpose of the study. Then, all population who wished to participate in the study were interviewed and screened. Written and informed consent was obtained from all participants.

Satisfied healthy non-smokers (current or past smokers defined as those who had smoked at least 100 cigarettes in life and/or had a previous lifetime exposure of > one pack-year of smoking) and participants without a history of any current airway or lung disease (breathlessness, cough, wheeze, ischemic heart disease, and rheumatic disorders) were included in the study. The subjects who were not eligible for a baseline spirometry test and those who reported respiratory symptoms including a cough, sputum, rhinorrhea, etc. within seven days prior to the examination were excluded from the study.

Sex (female, male), age (one decimal point), height (with a precision of 0.5 cm without shoes using an accurate stadiometer), weight (with a precision of 0.5 kg measured without a jacket, bag, veil (in women), wristwatch, and with empty pockets) were recorded.

### Spirometric measurements

The advanced Spirobank II device (MIR, Rome, Italy) used in this study, and FEV_1_, FVC, FEV_1_/FVC, and FEF_25–75%_ (forced expiratory flow averaged over the middle portion of FVC) were measured.

The spirometers were calibrated every morning and a minimum of three and a maximum of eight measurements were performed per subject. The measurements were made without the use of bronchodilators according to the American Thoracic Society/European Respiratory Society (ATS/ERS) recommendations^3^. The repeatability criterion was < 5% deviation from the second-highest value. From the three selected large values that were within 150 ml of each other, the largest measurement was chosen as the best.

### Quality control

The spirometry software provided feedback on the acceptability of the technique and repeatability. For spirometry according to the HUNT3/YoungHUNT3, curves were graded as A–F partly in line with a recent study by Hankinson et al.^18^. All curves graded as A–C were included in the study, i.e., at least two acceptable blows with a less than 150 mL difference. The inter-and intra-observer agreements showed excellent results.

### Sample size

According to the ERS/Global Lung Function Initiative (GLFI), representative samples of at least 300 subjects can be used for validation in groups not covered by the GLI equations^8^. Six hundred females and 300 males in different age groups (4–82 years old) were selected. The sample size for any center was calculated based on the proportion of population per regional municipality. Finally, by removing samples with grades D and F 622 subjects (204 males and 418 females) were fully eligible to enter the study.

### Analysis

Using the Excel macro for GLI^8^, reference values, lower limits of normal (LLN), Z-scores, and percentiles for FEV_1_, FVC, FEV_1_/FVC, and FEF_25–75%_ were calculated for each subject in the population. If the agreement between the observed values in the reference population and the GLI reference values is perfect, the mean Z-scores should ideally be zero, and the standard deviation (SD) should be one. According to the agreement reached by the GLI team and other studies validating these spirometric reference equations (SRE), a mean Z-score outside the range of ± 0.5 is considered clinically significant, corresponding to at least 5–6% difference in the specified lung function measurement^8,13,16,19,20^.

The mean values and standard deviations were calculated, and Z-score curve plots were drawn. Possible relationships between Z-scores and age, height, weight, and sex were examined using multiple linear regression models. If the GLI reference values are applicable, no such relationships exist.

LLN was defined as the lower fifth percentile in the distribution from which the GLI reference values are derived, as calculated by the GLI Excel macro, if not explicitly stated otherwise. The 90% limits of normality, which are expected to include 90% of the observations if the agreement is perfect, were defined as observations with GLI Z-scores within the − 1.645 to + 1.645 range^8^.

### Ethical approval and consent to participate

This article was the result of a research project approved by the National Institute for Medical Research Development (NIMAD) (code: 978931, 2019/05/28) and the Ethics Committee (code: IR.NIMAD.REC.1398.257). Written consent was obtained from all participants.

### Consent for publication

No personal information of the participants in the article was reported.

## Results

Between 16 July and 27 August 2019, 900 participants completed spirometry measurements. After exclusions, 622 participants (204 males and 418 females) aged 4–82 years met the selection criteria for the reference sample.

The mean (range) age of men and women was 38.34 (4–82) and 44.55 (4–80) years, respectively. In this sample, the mean height was 1.72 (0.08) m in males and 1.58 (0.08) m in females aged over 21 years. Thirty-nine (19.2%) men and 131 (31.4%) women had a BMI ≥ 30 kg/m^2^. The demographic and clinical characteristics of the participants are described in Table 1.

**Table 1:** Demographical and clinical characteristics of the reference population.

Variables	Total (620)	Females (414)	Males (206)	*P* value
Mean age (SD); year	42.29 (17.34)	45.54 (15.67)	37.78 (19.56)	< 0.001
Range	4–82	4–80	4–82	
Mean weight (SD); kg	70.73 (18.18)	68.61 (16.25)	74.88 (20.99)	< 0.001
Range	14–132.5	15–120	14–132	
Mean height (SD); cm	160.18 (14.21)	156.18 (11.09)	168.22 (16.27)	< 0.001
Range	101–193	101–120	109–193	
Mean BMI (SD)	27.17 (5.65)	27.84 (5.74)	25.8 (5.23)	< 0.001
Range	11.16–49.95	13.63–49.95	11.16–39.84	
Mean FEV1 (SD)	2.97 (0.94)	2.63 (0.65)	3.65 (1.04)	< 0.001
Range	(0.79–6.58)	0.79–5.01	0.86–6.58	
Mean FVC (SD)	3.61 (1.12)	3.20 (0.78)	4.42 (1.24)	< 0.001
Range	0.90–7.5	0.9–0.78	1.15–7.5	
Mean SVC (SD)	3.35 (1.17)	2.93 (0.85)	4.18 (1.26)	< 0.001
Range	0.54–7.24	0.34–6.9	0.91–7.24	
Mean FEV_1_/FVC (SD)	0.82 (0.069)	0.820 (0.70)	0.826(0.067)	0.878
Range	0.5–1.49	0.65–1.49	0.51–1.0	
Mean FEV_1_/VC (SD)	0.920 (0.358)	0.89 (0.42)	0.892 (0.169)	0.156
Range	0.342–8.82	0.413–8.82	0.34–1.64	
Mean FEF_25–75%_ (SD)	1.36 (0.457)	3.88 (1.29)	1.51 (0.523)	0.181
Range	0.46–2.22	0.49–8.13	0.55–2.22	

*BMI* Body Mass Index, *FEF*_*25–75%*_ forced expiratory flow at 25 and 75% of the pulmonary volume, *FEV*_*1*_ forced expiratory volume in 1 s, *FVC* forced expiratory vital capacity, *SD* standard deviation, *SVC* slow expiratory vital capacity, *VC* vital capacity.

The Caucasian GLI-2012 was applied to our population. Overall, the mean Z-scores of FEV_1_, FVC, and the FEV_1_/FVC ratio for males and females in the various age groups were higher than the Caucasian predicted values (range: 0.01 to 1.05) except for the FEV_1_/FVC in the age group under 21 years (range: − 1.11 to − 0.09).

**Figure 1:**
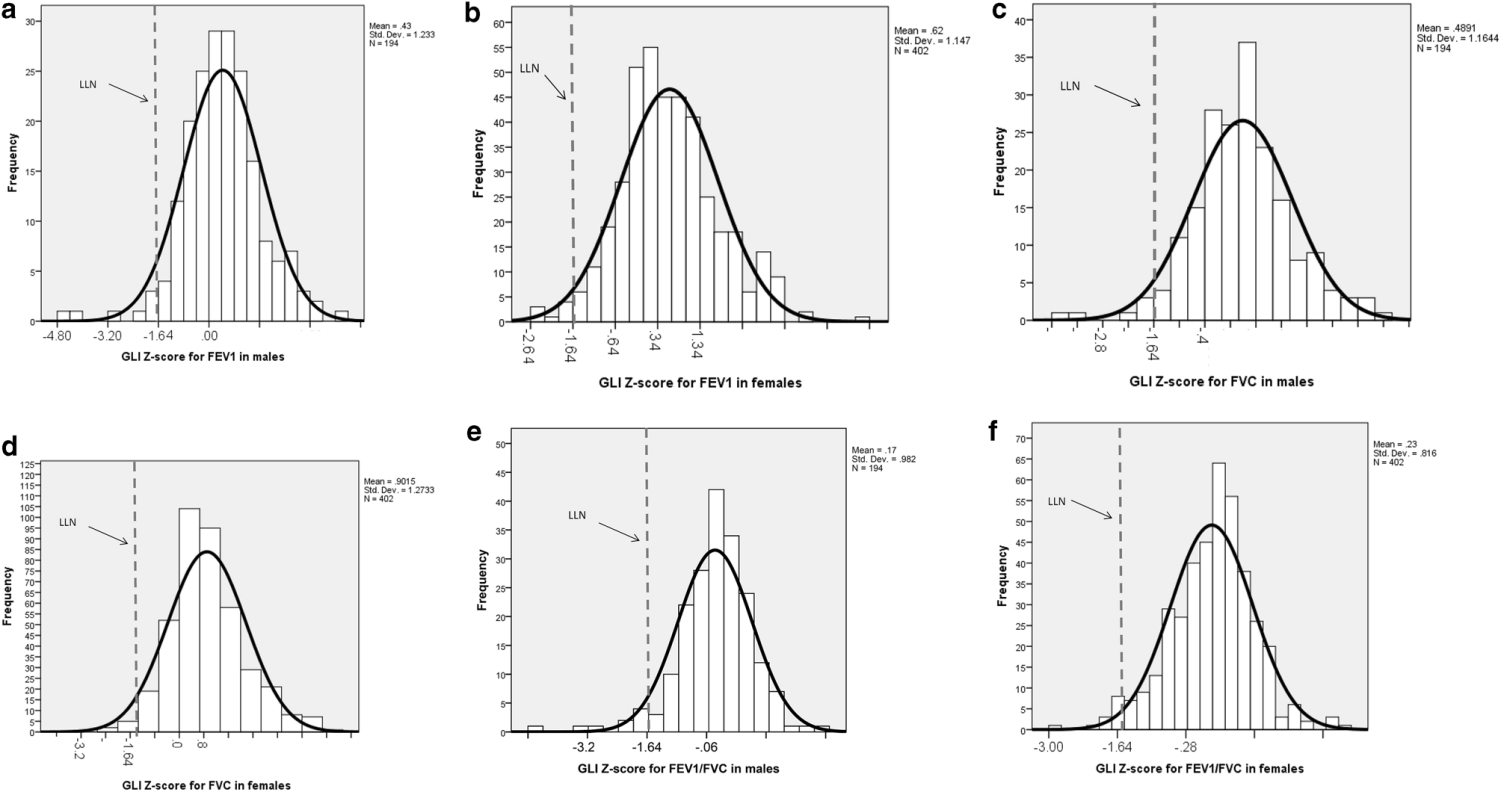
(**a**–**f**) Normal distribution curves of FEV1, FVC and FEV1/FVC based on observed mean GLI Z-score values in men and women.

The normal distribution curves of FEV_1_, FVC, and FEV_1_/FVC based on observed GLI Z-score mean and standard deviation values in men and women are presented in Fig. 1a–f.

The distribution of the Z-scores of FEV_1_, FVC, FEV_1_/FVC, and FEF_25–75%_ stratified by sex and age in the reference sample of healthy individuals is shown in Table 2.

**Table 2:** Mean GLI Z-scores for FEV_1_, FVC, the FEV_1_ /FVC ratio and FEF_25–75%_ by age group and sex (assumption test: compare means with zero).

Age group (Y)	Sex	n	FEV1			FVC			FEV1/FVC			FEF_25–75%_		
			Mean	SD	*P* value	Mean	SD	*P* value	Mean	SD	*P* value	Mean	SD	*P* value
< 10	Male	13	0.52	**0.62**	0.010	0.72	**0.63**	0.001	− 1.11	1.22	0.001	**− 0.11**	**0.82**	0.637
	Female	20	**0.40**	**0.90**	0.061	0.62	**0.79**	0.003	− 1.2	1.08	0.003	− 0.59	**0.62**	0.000
10–21	Male	38	**0.27**	1.29	0.206	**0.39**	1.3	0.065	**− 0.09**	1.01	0.065	**− 0.09**	1.02	0.595
	Female	15	**0.37**	1.09	0.203	0.50	**0.69**	0.013	**− 0.32**	1.22	0.013	**− 0.19**	1.46	0.617
22–29	Male	21	**0.16**	**0.79**	0.357	**0.29**	**0.71**	0.075	**0.10**	**0.52**	0.075	**− 0.44**	**0.94**	0.047
	Female	23	**0.14**	**0.89**	0.446	**0.42**	1.02	0.060	**0.01**	1.04	0.060	− 0.66	1.00	0.004
30–39	Male	43	**0.46**	1.27	0.022	0.54	1.22	0.006	**0.23**	**0.69**	0.006	**− 0.11**	1.1	0.532
	Female	84	0.50	1.24	0.000	0.89	1.29	0.000	**0.06**	**0.67**	0.000	− 0.58	0.86	0.000
40–49	Male	25	0.65	0.85	0.001	0.65	**0.93**	0.002	**0.42**	**0.63**	0.002	**− 0.07**	**0.77**	0.646
	Female	99	0.57	1.16	0.000	0.91	1.2	0.000	**0.28**	**0.77**	0.000	**− 0.49**	1.08	0.000
50 − 59	Male	29	0.59	1.09	0.006	0.62	**0.97**	0.002	**0.42**	**0.88**	0.002	** − 0.22**	**0.89**	0.192
	Female	106	0.83	1.11	0.000	1.05	1.48	0.000	**0.45**	**0.78**	0.000	** − 0.41**	**0.91**	0.000
60 − 69	Male	19	0.51	1.02	0.041	**0.30**	**0.9**	0.161	0.95	**0.83**	0.161	**0.19**	1.08	0.438
	Female	63	0.72	1.15	0.000	1.02	1.18	0.000	**0.32**	**0.76**	0.000	− 0.53	**0.86**	0.000
> 70	Male	16	**0.36**	2.23	0.531	**0.48**	2.02	0.361	− 0.61	1.65	0.361	**0.0**	1.28	0.989
	Female	8	**0.36**	**0.82**	0.260	**0.06**	**0.66**	0.805	**0.07**	**0.98**	0.805	**0.09**	1.08	0.830

*GLI* Global Lung Function Initiative, *FEF*_*25–75%*_ forced expiratory flow at 25 and 75% of the pulmonary volume, *FEV*_*1*_ forced expiratory volume in 1 s, *FVC* forced expiratory vital capacity, *P* values by independent samples T-test, *SD* standard deviation, *Y* years.

Means with statistical significant *P*(*p* value) turned into bold.

The FEV_1_ Z-score was smaller than 0.5 in men and women aged 10–21, 22–29, and 30–39 years; however, its standard deviation was often above 1. Moreover, this value was below 0.5 in girls under 10 years old and men 40–49 years old and over 70. The FEV_1_ Z-score was not different from zero (by one-sample t-test analysis) in the age groups 10–29 and over 70 years in both genders (*P* > 0.05) (Table 2).

The FVC Z-score exceeded the predicted values (0.5) across age groups < 10, 30–39, 40–49, and 50–59 years in both genders, but it was below 0.5 in individual aged 10–21 (males), 22–29 (both gender), 60–69 (males), and > 70 (both gender) years old (Table 2).

The Z-score of FEV_1_/FVC was below 0.5 in all age groups except for the age group under 10 (both genders) and 60–69 (males) years (Table 2).

The mean Z-score of FEF_25–75%_ was between 0 and − 0.5 in males in all age groups; the same finding was found in women aged 10–21, 40–49, 50–59, and 70–84 years (Table 2).

In the age group over 21 years, the Z-score of FEV_1_/FVC was below the LLN in seven men (3.43%) and eight women (2.01%). However, these values were significantly higher in six boys (46.2%) and eight girls (40.0%) under 21 years old.

Z-score of FEV_1_/FVC less than LLN was zero in women over the age of 60 and in men aged 22–39 and 60–69 years. It was also less than 5% in women aged 50–59 and men aged 30–59 years (1–3.4%).

Totally, the FEV_1_/FVC Z-score was above the upper limit of normal in 20 (9.8%) men and 69 (16.5%) women (ULN > 1.64).

The mean Z-score of FEV_1_/FVC above the upper limit of normal (ULN, > 1.64) ranged between 0% (age group 22–29 years) and 25% (over 70 years) in men and between 0% (age group 10–21 and over 70 years) and 28.3% (age group 50–59 years) in women.

The FEV_1_ Z-score was within the 90% limits of normality (− 1.64 to + 1.64) in 81.3% of the observations (83.7% in males and 80.4% in females). The corresponding figure was 78.9% for FVC (84.5% in males and 76.1% in females) and 93.3% (90.2% in males and 94.8% in females) for the FEV_1_/FVC ratio in the age group over 21 years.

The Z-scores of FEV_1_, FVC, FEV_1_/FVC, and FEF_25–75%_ were analyzed according to age, height, weight, and gender using a linear regression model.

**Figure 2:**
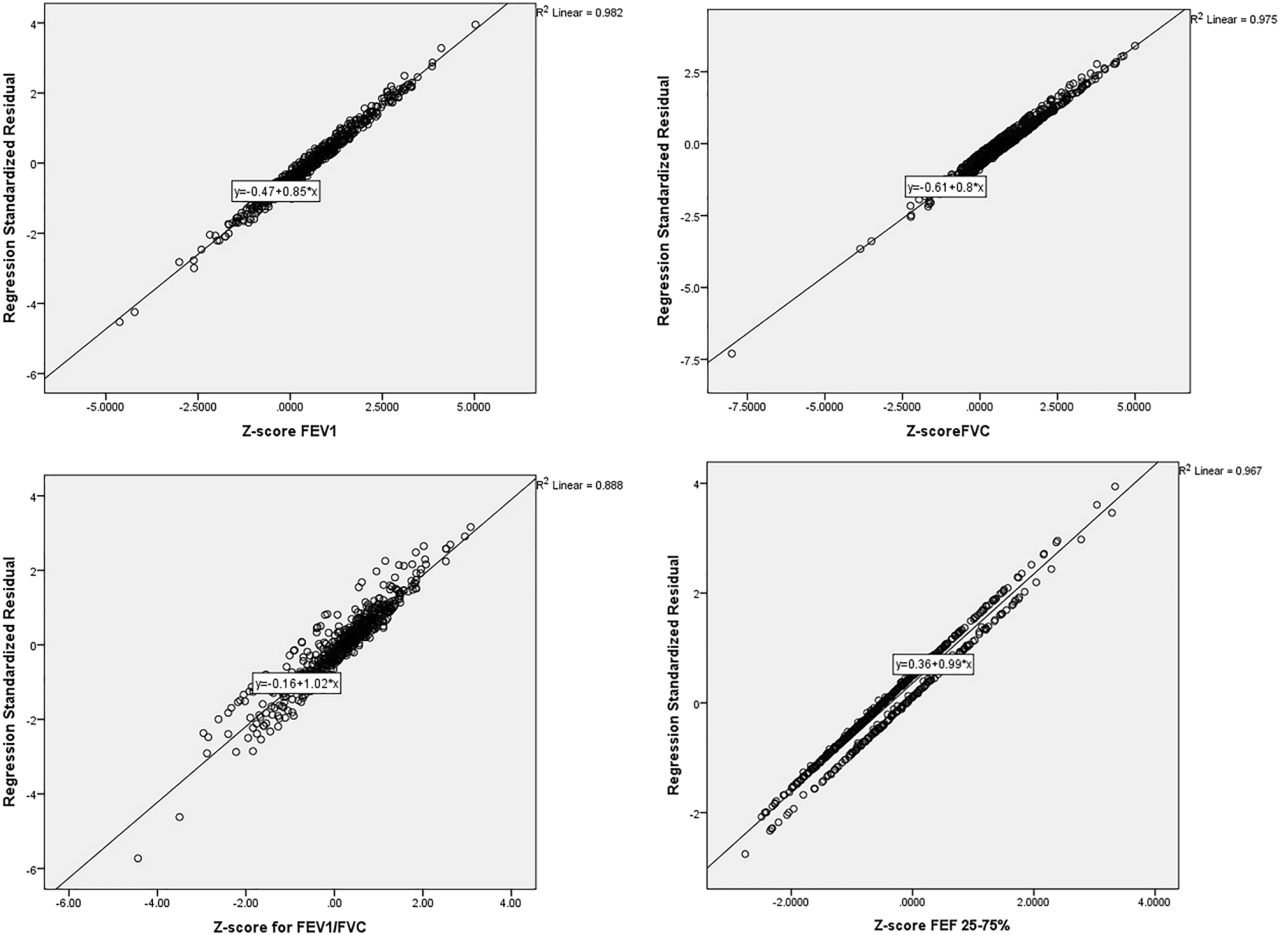
(**a**–**d**) Association of GLI2012 mean Z-score values of FEV1, FVC, FEV1/FVC, and FEF_25–75%_ with gender, age, and anthropometric variables according to multiple linear regression.

Age, weight, and height (but not gender) had an impact on the FEV_1_/FVC Z-score in univariate regression (*P* < 0.05). In multiple linear regression (in the presence of height, weight and age as variables with *P* < 0.2 in univariate linear regression) height and age remained associated (B-coefficient = 0.012 and 0.007; *P* = 0.008, *P* = 0.001 respectively) with the FEV_1_/FVC Z-score (Fig. 2a). There was a significant association between age and FEV_1_ Z-score (B-coefficient = 0.007; *P* = 0.011). In the multiple linear regression (in the presence of age, gender and height), age remained statistically significant (B-coefficient = 0.008; *P* = 0.006) (Fig. 2b).

There was a significant association between gender and FVC Z-score (B-coefficient = 0.389; *P* < 0.001) in univariate linear regression; this association was maintained in the multiple regression model too (B-coefficients = 0.346; *P* = 0.004) (Fig. 2c).

Height and gender had an effect on the FEF_25–75%_ Z-score (B-coefficients =  − 0.371 and 0.006; *P* < 0.001 and *P* = 0.021, respectively) in univariate linear regression. In the multiple linear regression model, gender had a significant relationship with the FEF_25–75%_ Z-score (B-coefficient =  − 0.358; *P* = 0.001) (Fig. 2d).

The prevalence of COPD defined by spirometry based on the fixed ratio (FR) criterion increased with age from 0.8% in the age group 30–39 years to 16.7% in the age group > 70 years (P_for linear by linear association_ < 0.001). The prevalence of COPD according to the LLN criterion did not follow a specific trend (P_for linear by linear association_ = 0.749). There was a 98.13% agreement between FR and LLN method (Fleiss' kappa coefficient = 0.58, *P* < 0.001). The prevalence of COPD based on FR and LLN according to age and sex is presented in Table 3.

**Table 3:** Prevalence of COPD based on FR (FEV1/FVC < 0.70) and LLN (FEV1/FVC < LLN) criteria by age and sex (n (%)).

Age groups	Total (589)		Males (191)		Females (398)	
	FR	LLN	FR	LLN	FR	LLN
< 10 (33)	0	14 (42.4)	0	6 (46.2)	0	8 (40.0)
10–21 (53)	0	4 (7.5)	0	2 (5.3%)	0	2 (13.3)
22–29 (44)	0	2 (4.5)	0	0	0	2 (8.7)
30–39 (127)	1 (0.8)	1 (0.8)	0	0	1 (1.2)	1 (1.2)
40–49 (124)	1 (0.8)	1 (0.8)	0		1 (1.0)	1 (1.0)
50–59 (135)	2 (1.5)	3 (2.2)	1 (3.4)	1 (3.4)	1 (0.9)	2 (1.9)
60–69 (82)	4 (4.9)	0	0	0	4 (6.3)	0
> 70 (24)	4 (16.7)	4 (16.7)	4 (25.0)	4 (25.0)	0	0

*FR* fixed ratio (FEV1/FVC < 0.7), *LLN* lower limit of normal (FEV1/FVC < LLN).

## Discussion

This study was the first study performed on 622 healthy non-smoker Iranian children and adults to evaluate the use of the GLI-2012 reference values to interpret FEV_1_, FVC, FEV_1_/FVC and FEF_25–75%_.

When applying the GLI reference values to the reference population, the Z-scores were always closer to zero in men compared to women. The mean Z-score (SD) values of FEV_1_/FVC and FEF_25–75%_ (in both sexes) were reasonable, although not perfectly, normally distributed but not centered on 0 (0.11 (1.03) and 0.17 (0.88) for FEV_1_/FVC, and − 0.11 (0.99) and − 0.49 (0.96) for FEF_25–75%_ in males and females, respectively. Although the Z-scores of FEV_1_ and FVC were below 0.5 in men, they were between 0.5 and 1 in women. In addition, the SD of all measurements was below 1.5. The Z-scores of spirometry indices of some countries^1,6,10–13,16,17^ are shown in Table 4.

**Table 4:** Z-scores for spirometry indices of some countries (source populations: never-smokers without respiratory symptoms).

References	Design, date	Country (race)	Sample size	Male (%)	Age range	FEV1 (SD)	FVC (SD)	FEV1/FVC (SD)	Consistency with GLI-2012
Our study	Cross-sectional, 2019	Iran	204	32.8	4–82	0.44 (1.21)	0.49 (1.14)	0.11 (1.03)	Not perfect
			418	67.2	4–80	0.61 (1.14)	0.89 (1.26)	0.17 (0.88)	No
Blake et al.^10^	Cohort	Australia	930	49.7	3–25	− 0.53 (0.85)	− 0.47 (0.8)	− 0.12 (0.75)	Yes
Zhang et al.^17^	NHANES data, 2011	Asian American	567	44.0	6–79	< − 0.5 (< 1.5)	< − 0.5 (< 1.5)	< − 0.5 (< 1.5)	Yes
Abdullah et al.^16^	Cross-sectional, 2007	Malay	11,876	17.0	6–79	− 1.77 (0.86)	− 2.32 (0.88)	1.34 (1.02)	No
		Chinese	13,619	20.0	6–79	− 1.21 (0.86)	− 1.03 (0.95)	1.14 (1.08)	
		Indian	4786	27.0	6–79	− 2.12 (0.85)	− 1.21 (0.87)	1.54 (1.08)	
Backman et al.^13^	Cohort, 2006**	Sweden	501	52.0	20–69	0.21 (0.91)	0.35 (0.32)	− 0.25 (0.85)	Not perfect
Langhammer et al.^1^	Cohort, 2003–2008	Norwegian	11,163	52.3	12–19	0.0 (1.05)	0.009 (1.03)	− 0.11 (0.89)	Yes
				42.4	20–90	0.13 (0.95)	0.19 (0.89)	− 0.11 (0.8)	
Ben Saad et al.^6^	Cross-sectional	Tunisia	489	80.4	18–60	− 0.55 (0.87)	− 0.62 (0.86)	0.1 (0.73)	No
Huls et al.^11^	Cohort 1985–2013	Germany	299	0.0	52–83	− 0.11 (0.90)	0.07 (0.81)	− 0.35 (0.79)	Yes
Hulo et al.^12^	Cross-sectiona^1^*	France	904	0.46	40–65	0.01 (1.11)	0.18 (1.0)	− 0.32 (0.87)	Yes
			1067	0.54	40–65	0.51 (1.0)	1.3 (1.11)	− 0.25 (0.85)	Yes

*FEV*_*1*_ forced expiratory volume in 1 s, *FVC* forced expiratory vital capacity, *SD* standard deviation.

*Source population: Healthy population but regardless of smoking status.

**The GLI reference values are superior, but not perfect, for Swedish adults.

A number of studies have found that the use of the GLI equation was ideal in their population, including Spain where the Z-score (mean) of each parameter was close to 0 with a maximum variance of ± 0.5^21^. Other populations in which the use of GLI-2012 equation has been approved include Norway, Australia, Germany, and America (Asian–Americans)^1,10,11,17^. Although a review of the Z-score of spirometry values in Sweden showed that these values were relatively appropriate, the authors emphasized the lack of equation matching^13^. Moreover, although it was reported that the GLI equation could be used in the French population aged 40–65 years, the standard Z values appeared be relatively large in women (FEV_1_ = 0.51, FVC = 1.3). Therefore, it seems that in approving or rejecting the fit of the equation with the source population, the authors’ opinion also matters such that some studies were strict while some were not (Table 4). In general, the GLI equation did not fit the source population in studies conducted in Tunisia, China, Malesia, India, and Sweden.

Comparison of the obtained values (FEV_1_, FVC, FEV_1_/FVC, and FEF_25–75%_ Z-scores) with the predicted values according to the age group confirmed the fit of lung function parameters (except FEV/FVC for men) for subjects aged 10–21, 22–29 and 70–84 years.

The FEV_1_/FVC Z-score was always under 0.5 in all age groups except males and females under 10 years and men aged 60–84 years.

A few studies tried to standardize the spirometric measurements in certain age groups including children and adolescents. Some of these studies confirmed the fit of GLI-2012 reference values in their community. However, some other studies emphasized that reference equations did not match the spirometric data in their children. In a study (2013) conducted in white, black, and South-Asian schoolchildren aged 5–11 years in London, GLI-2012 reference equations properly fitted spirometric data in white and black races. These values were fit for South Asian children based on the Southeast Asian equation^22^. Among 712 healthy urban-dwelling 7–13 year-old Zimbabwean schoolchildren, the mean GLI2012 Z-scores of FVC, FEV_1_, FEV_1_/FVC, and maximal-mid expiratory flow (MMEF) were measured using different ethnic GLI 2012 modules. The mean African-American GLI 2012 Z-scores were within 0.5 Z-scores from zero for all the spirometry variables; however, the Z-score SD for the FEV_1_/FVC ratio was ≥ 1, indicating more variability than the reference population, thus affecting the performance of the African-American GLI2012 LLN in this population^23^.

Chang et al. studied the age group 5–18 years in Taiwan in 2019 and provided evidence that the GLI-2012 reference equations did not properly match the spirometric data in the current Taiwanese pediatric population, indicating an urgent need for an update of the GLI reference values by the inclusion of more data of non-Caucasian descent^24^. Moreover, in the study by Jones et al. in 2020, the equations currently in use in Brazil seemed to underestimate the lung function of Brazilian children aged 3–12 years^25^. The GLI-2012 reference values for spirometry were appropriate for healthy, well-nourished African school children in Angola, DR Congo, and Madagascar, but the lower limit of normal needed adjustment^20^.

In this study, lung function tests in 33 children (13 boys and 20 girls) aged 4–10 years were performed with acceptable quality of grades A to C. The FVC, FEV_1_, FEV_1_/FVC, and FEF_25–75%_ values were measured, and almost all values (except for FEV_1_ in girls and FEF_25–75%_ in boys) were above or below the expected values (> + 0.5 or <  − 0.5); however, the standard deviation was estimated to be less than one in all cases except for FEV_1_/FVC. Z-scores, FEV_1_/FVC and FEF_25–75%_.

The percentage of the population with the FEV_1_/FVC value below LLN in the age group over 21 years (in both genders) was appropriate (3.43% in men and 2.1% in women) and was less than 5%. In the age group 10–21 years, the percentage of population with the FEV_1_/FVC value below LLN was relatively appropriate in men (5.03%) but was higher than expected in women (13.3%). These values were extremely high (42.4% in boys and 40% in girls) in the under 10 age group.

The Z-scores derived from the GLI-2012 SRE showed significant association with gender, age, and anthropometric variables. In multiple linear regression, height and age had positive associations with FEV_1_/FVC (B-coefficient = 0.01 and B-coefficient = 0.007), age had a positive association with FEV_1_ (B-coefficient = 0.008), and gender had a relatively strong relationship with FVC (B-coefficient = 0.346) and FEF_25–75%_ (B-coefficient = 0.358).

### Limitation of the study

The number of sample sizes in age groups under 10 years old (13 cases) in boys and over 70 years old (8 cases) in women was under 15 cases.

## Conclusion

The GLI reference values are not perfect for the Iranian population, especially in children under 10 years old and females. However, the Z-score of FEV1/FVC matched the predicted value in almost all age groups (except children below 10 years of age). GLI reference values were appropriate for subjects over 21 years, but their use would overestimate the prevalence of airway obstruction in the Iranian population under 21 years. The results of linear regression models showed that age, height and, gender were crucial for establishing prediction equations of four spirometric measurements, i.e., FEV1/FVC, FVC, FEV1, FEF_25–75%._

## Data Availability

Data available on request from the authors.
